# Development of Brain Structural Networks Over Age 8: A Preliminary Study Based on Diffusion Weighted Imaging

**DOI:** 10.3389/fnagi.2020.00061

**Published:** 2020-03-10

**Authors:** Zhanxiong Wu, Yun Peng, Sudhakar Selvaraj, Paul E. Schulz, Yingchun Zhang

**Affiliations:** ^1^School of Electronic Information, Hangzhou Dianzi University, Hangzhou, China; ^2^Department of Biomedical Engineering, University of Houston, Houston, TX, United States; ^3^Louis A. Faillace, MD, Department of Psychiatry and Behavioral Sciences, The McGovern Medical School of UT Health Houston, Houston, TX, United States; ^4^Department of Neurology, The McGovern Medical School of UT Health Houston, Houston, TX, United States

**Keywords:** magnetic resonance imaging, diffusion weighted imaging, ensemble average propagator, structural network, brain development

## Abstract

Brain structural network changes provide key information about the aging process of the brain. Unfortunately, there has yet to be a detailed characterization of these structural networks across different age groups. Efforts to classify these networks have also been hampered by their reliance on technically limited traditional methods, which are unable to track multiple fiber orientations within a voxel and consequently are prone to false detection and artifacts. In this study, a newly developed Ensemble Average Propagator (EAP) based probabilistic tractography method was applied to construct a structural network, with the strength of the link between any two brain functional regions estimated according to the alignment of the EAP along connecting pathways. Age-related changes in the topological organization of human brain structural networks were thereby characterized across a broad age range (ages 8–75 years). The data from 48 healthy participants were divided into four age groups (Group 1 aged 8–15 years; Group 2 aged 25–35 years; Group 3 aged 45–55 years; and, Group 4 aged 65–75 years; *N* = 12 per group). We found that the brain structural network continues to strengthen during later adolescence and adulthood, through the first 20–30 years of life. Older adults, aged 65–75, had a significantly less optimized topological organization in their structural network, with decreased global efficiency and increased path lengths versus subjects in other groups. This study suggests that probabilistic tractography based on EAP provides a reliable method to construct macroscale structural connectivity networks to capture the age-associated changes of brain structures.

## Introduction

Human brain structural networks are functionally modular and connect effectively through neural bundles to meet the needs for complex cognitive tasks (Schmahmann et al., 2007; Lerch et al., 2017). This neural fiber connectivity enables the communication between the various regions of the brain (Bassett et al., 2011) and its integrity is pivotal for individual health. Due to the development of non-invasive imaging technologies, such as diffusion-weighted imaging (DWI), our knowledge of these structural pathways have vastly improved. DWI characterizes structural connectivity networks across brain regions *in-vivo* by calculating the number of streamlines or the probability of connections (Frank, 2002; Tuch et al., 2002; Anderson, 2005; Maier-Hein et al., 2017). The demonstrated connectivity patterns can then be assessed through graph-based analyses that outline the complex structural substrates of cognition (Betzel et al., 2014). This approach has been effectively employed to identify densely interconnected structural hub regions that are critical to efficient neuronal signaling and communication (van den Heuvel and Sporns, 2013).

Aging has been recognized as a significant factor affecting brain functions. Specifically, a growing body of evidence suggests that brain structure is altered as age increases. Although the fundamental cause of these age-related white matter changes has yet to be fully understood, current theories tie them to changes in the axonal diameter and myelination, synaptic pruning, and modification (Onoda et al., 2012; Dennis and Thompson, 2014; Geerligs et al., 2014; Huang et al., 2015; Grady et al., 2016). According to the “early-in-late-out” hypothesis, increases in fiber tract white matter density and axonal myelination are important for cognitive development during childhood and adolescence (Paus et al., 1999). In contrast, demyelination and a loss of nerve fibers contribute to cognitive decline (Peters, 2002). Despite these early hypotheses and studies, available data describing age-related changes in structural connectivity remains lacking (Damoiseaux, 2017).

While limited, currently available evidence suggests that old age is associated with lower connectivity and lower local efficiency (Damoiseaux et al., 2007; Gong et al., 2009; Burzynska et al., 2010; Otte et al., 2015). These studies, however, address coarsely divided age subgroups. In addition, recent evidence from Zhao et al. (2015) also suggests that the age-related trajectories of local and global structural network efficiency changes are non-linear. The use of large age ranges may then combine subjects at different stages, complicating the study of age-related structural connectivity changes that would otherwise provide useful insights for patient diagnosis and management.

Several methods have been developed to reconstruct structural connectivity networks. Previous studies have relied heavily on diffusion tensor imaging (DTI) methods to trace white matter connections (Gong et al., 2009; Betzel et al., 2014; Otte et al., 2015; Zhao et al., 2015). Conventional DTI, however, struggles to resolve complex fiber populations when they occur within a DWI voxel, as is the case when tracts cross, branch, merge, or kiss (Tuch, 2004; Hess et al., 2006; Poupon et al., 2008). As approximately one- to two-thirds of DWI voxels contain multiple fiber populations (Duarte-Carvajalino et al., 2014), structural connectivity networks reconstructed based on diffusion tensor may deviate largely from real situations. It is then necessary to develop methods capable of parsing these complex white matter structures.

In this study, age-related changes in structural connectivity were investigated using a novel Ensemble Average Propagator (EAP)-based probabilistic tractography method. Unlike DTI or orientation distribution function methods, EAP preserves the radial part of the diffusion signal and thus may accurately identify the crossing orientations of neural fascicles within a white matter voxel (Fick et al., 2014; Paquette et al., 2015). The aim of this study was to evaluate the novel EAP approach for reconstructing structural connectivity networks, using a healthy subject dataset that covered a wide range of ages.

## Materials and Methods

### DWI Dataset

**FIGURE 1:**
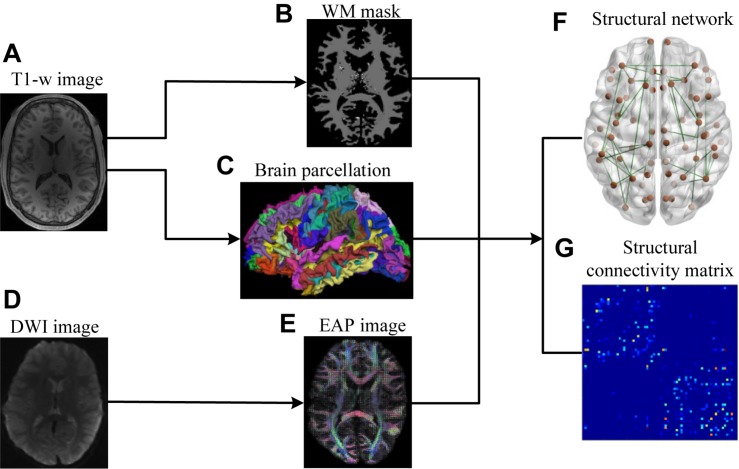
Flowchart for the construction of structural connectivity networks based on EAP. **(A)** High-resolution T1-weighted MRI images. **(B)** White matter mask. **(C)** Cerebral cortex parcellation from T1-weighted MRI images using FreeSurfer. **(D)** DWI images. **(E)** EAPs constructed with SPFI. **(F)** Structural connectivity Networks. **(G)** Structural connectivity matrix. Before SC network construction, **(B,C)** are co-registered into DWI B0 native space in FreeSurfer.

The lifespan datasets publicly available from the Human Connectome Project (HCP) (Marcus et al., 2011; Van Essen et al., 2013) and the OASIS3 database (Longitudinal neuroimaging, clinical, and cognitive dataset for normal aging and Alzheimer’s disease^1^) were used in this study. Datasets included 48 subjects aged 8–75 years, organized into 4 age groups (Table 1). HCP subjects (34) underwent an abbreviated scan protocols similar to that used for the WU-Minn young adult HCP study (Feinberg et al., 2010). Data for each subject was collected using a 3T General Electric MR scanner, with a whole-body radiofrequency coil for signal excitation and a quadrature 8-channel brain coil for the reception. The HCP acquisition protocol consisted of: (1) high resolution 3D T1-weighted SPGR sequence, with TR/TE/flip angle of 9.1 ms/4.1 ms/108, acquisition matrix size = 256 × 256 × 320, and slice thickness = 1 mm; (2) DWI using a single-shot spin-echo echo-planar sequence, including 91 non-collinear encoding directions with b values of 1000, 2000, 3000 s/mm^2^, TR/TE = 12700/88.3 ms, acquisition matrix size = 144 × 168 × 111, slice thickness = 2.4 mm, and voxel size = 2.4 mm × 2.4 mm × 2.4 mm, along with six additional images with no diffusion sensitization *b* = 0 s/mm^2^ (b0, non-diffusion-weighted images). The remaining 14 subjects from the OASIS3 dataset were scanned using a Siemens TIM Trio 3T MRI scanner with 16-channel head coils and the scan protocols that included: (1) high-resolution 3D T1-weighted GR IR with TR/TE/flip angle = 2.4 s/3.16 ms/8, acquisition matrix size = 256 × 256 × 176; (2) DWI using a single-shot spin-echo echo-planar sequence with TR/TE/flip angle = 9.9 s/102 ms/90, *b*-values = 600, 800, 1000 s/mm^2^, acquisition matrix size = 96 × 96 × 60, slice thickness = 2 mm, and voxel size = 1.98 mm × 1.98 mm × 2 mm, wherein diffusion-weighted gradients were applied along 25 directions with one b0 image. The overall process for scanning and data analysis is summarized in Figure 1.

**TABLE 1:** Lifespan datasets from HCP and OASIS3 database, including four age groups.

Age (years)	*N*	Gender
8-15	12	8 Female, 4 Male
25-35	12	5 Female, 7 Male
45-55	12	5 Female, 7 Male
65-75	12	6 Female, 6 Male

### Reconstruction of the Structural Connectivity Network

Brain parcellation and reconstruction were performed using FreeSurfer (stable version 6.0.0)^2^ (Fischl, 2012). Parcellations for each subject were generated in the native space, based on the collected high-resolution T1-weighted MRI images. The cerebral cortex was parcellated into 68 functional regions, 34 for each hemisphere (Desikan et al., 2006). These regions were considered as the nodes in a structural connectivity graph. Lastly, the corresponding atlas was affine registered to the DWI native space for each subject. The 68 cortical regions used to construct brain SC networks are list in Appendix Table A1.

The DWI dataset was first denoised following the procedure outlined by Wu et al. (2019b). The topup and eddy_openmp FSL 6.0 commands (Ubuntu Linux 16.04) were then used to correct eddy distortion and motion artifact (Andersson et al., 2003; Andersson and Sotiropoulos, 2015, 2016). Briefly, these corrections were performed by first performing an affine alignment of each DWI image to the b0 image. Next, EAPs were estimated from multi-shell DWI samples using spherical polar Fourier imaging (SPFI) (Cheng et al., 2010). According to a spherical harmonics expansion, EAPs were calculated along 726 directions evenly distributed about a spherical shell (Hess et al., 2006). Diffusion orientations contained within a white matter voxel were then extracted by detecting the local EAP peaks (Wu et al., 2018), which coincide with neural fiber tracts. Probabilistic fiber tracking was then performed to obtain the connection weights between different cortical regions (Iturria-Medina et al., 2007). Under angular constraints, the deterministic path planning algorithm was subsequently used to find all reasonable pathways between the WM voxels belonging to different ROIs. A train of consecutive WM voxels along each of the identified pathways was thereby determined, and the connection strengths of these pathways were computed by integrating their EAP alignment over a solid angle. The connection strength of each pair of WM voxels was then assigned as the connection strength with the largest connection possibility. Finally, the connection strength between each ROI pair was calculated as the sum of the connection probabilities for each pair of WM voxels within the ROIs (Wu et al., 2019a). The resulting link strength depended on the alignment of the EAP along connecting pathways, with higher strengths indicating better alignment. Evidence suggests that the link strengths determined by EAP fields may provide a more robust and suitable measure for structural connectivity network analysis than diffusion tensor and orientation distribution function (Assemlal et al., 2007; Cheng et al., 2010). To ensure that path propagations were anatomically realistic, we imposed a 90° maximum curvature threshold between every two successive path steps (Iturria-Medina et al., 2007, 2008; Sotiropoulos et al., 2010). This fiber tracking procedure yielded adjacency matrices whose elements represented the connection probabilities between each pair of parcellated cortical regions of interest.

Network graphs were created that consisted of a series of nodes connected by edges to interpret the generated adjacency matrices. Each node within a network graph represented a cortical region of interest, and the edges connecting them were assigned weights according to their determined link strength. In this study, each brain region was selected as the seed region, and its connectivity strength to each of the other 67 regions was calculated. Thus, for each subject, a 68 × 68 weighted, undirected network graph was constructed.

### Structural Connectivity Network Topological Analysis

A number of network metrics were adopted to quantify the topological features of the generated network graphs, including global efficiency *E*_*glob*_, local efficiency *E*_*loc*_, clustering coefficient *C*_*p*_, regional efficiency *E*_*nodal*_, and the small-world parameters γ and λ (Cao et al., 2013). After adjacency matrices were acquired and thresholded to remove weak connections, the enumerated network metrics were computed for each age group using the GRETNA (Wang et al., 2015) and BCT toolboxes (Rubinov and Sporns, 2010). Of particular interest here are the “small-world” parameters, which estimate the efficiency of information transfer within a defined network structure. An ideal, small-world network described by these parameters should feature a minimum path length between any pair of nodes equal to that of a comparable random network, with greater local interconnectivity or cliquishness (He et al., 2007). Lastly, the nodal efficiency metric, *E*_*nodal*_, was used to describe regional properties. The mathematical equations for these measures are provided in Table 2, and detailed descriptions of each are provided by Cao et al. (2013). The weights were thresholded from 0.05 and 0.21 in intervals of 0.02 to remove spurious connections. The information from these serial measurements was integrated to calculate the area under the curve (AUC) for each metric, such that the resultant AUC values summarized the topological organization of the brain structural connectivity networks, independent of a single threshold selection. To identify the differences across age groups, we performed Kruskal–Wallis tests on the lifespan subjects. Finally, quadratic regression using least square fitting was performed to fit the AUC values of each metric.

**TABLE 2:** Definitions of structural connectivity network metrics.

Network metrics	Description
$E_{g\,l\,o\,b}\,\left(G\right)=\frac{1}{N\,\left(N-1\right)}\,\sum_{i\neq j\in G}\frac{1}{L_{i\,j}}$	*L*_*ij*_is the shortest path length between node i and j in G.
$E_{l\,o\,c}\,\left(G\right)=\frac{1}{N}\,\sum_{i\in G}E_{g\,l\,o\,b}\,\left(G_{i}\right)$	*G*_*i*_denotes the subgraph composed of the nearest neighbors of node i.
$L_{p}\,\left(G\right)=\frac{1}{N\,\left(N-1\right)}\,\sum_{i\neq j\in G}L_{i\,j}$	*L*_*ij*_ is the shortest path length between node i and j. This function quantifies the ability for information to be propagated in parallel.
$C_{p}=\frac{1}{N}\,\sum_{i\in G}C\,\left(i\right)$	*C*_*p*_ is the average of the clustering coefficient over all nodes, which indicates the extent of local interconnectivity or cliquishness in a network.
$\gamma =C_{p}^{S\,C}\,/\,C_{p}^{r\,a\,n\,d}$	$C_{p}^{r\,a\,n\,d}$ is the mean*C*_*p*_of 100 matched random networks. $C_{p}^{S\,C}$ is the clustering coefficient empirical SC network.
$\lambda =L_{p}^{S\,C}\,/\,L_{p}^{r\,a\,n\,d}$	$L_{p}^{r\,a\,n\,d}$ is the mean*L*_*p*_of 100 matched random networks. $L_{p}^{S\,C}$ is the shortest length of the SC networks.
$E_{n\,o\,d\,a\,l}\,\left(i\right)=\frac{1}{N-1}\,\sum_{i\neq j\in G}\frac{1}{L_{i\,j}}$	*E*_*nodal*_(*i*) measures the average shortest pathway length between a given node *i* and all of the other nodes in the network.

### Age-Related Changes

To further investigate age-associated structural connectivity differences, the group-wise structural connectivity hub regions were identified for each group. These are defined as regions that critically enable efficient neuronal signaling and communication (van den Heuvel and Sporns, 2013), integrating information to support complex cognitive functions. Network nodes were considered to be brain hubs if their nodal efficiency *E*_*nodal*_ was at least one standard deviation greater than the average nodal efficiency of the network. Group-wise backbone links were also extracted from structural connectivity networks, which were formed based on the rich club connections for high-capacity brain communication (van den Heuvel et al., 2012).

## Results

**FIGURE 2:**
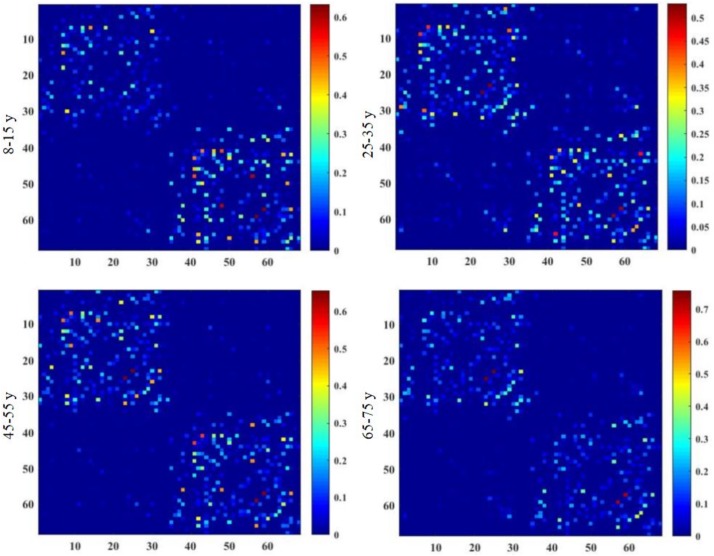
Group-averaged structural connectivity maps of HCP lifespan subjects, which are 68 × 68 symmetric matrices. The subjects are arranged into four groups by age, including 8–15, 25–35, 45–55, and 65–75 years groups. In the maps, self-connections are excluded. The numbers on the axes of connectivity matrix are the indexes of parcellated regions.

Brain structural connectivity networks were constructed for 48 subjects using probabilistic EAP-based tractography, including 68 nodes (Appendix Table A1). Figure 2 shows the consensus structural connectivity maps for each age group. These adjacency matrices were symmetric, with self-connections excluded. The values in these maps were normalized to [0, 1], with higher values indicating stronger anatomical connectivity. Based on these adjacency matrices, group-wise topological properties were examined to find the structural connectivity differences between age groups.

### Network Metrics

**FIGURE 3:**
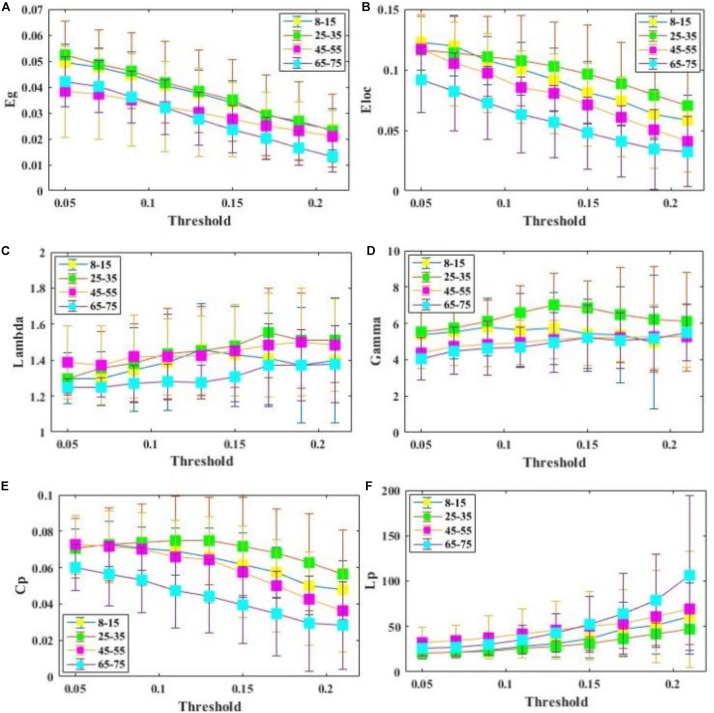
Comparison of global and regional network metrics across 4 age groups. **(A)** Global efficiency *E*_*glob*_, and it measures the global efficiency of parallel information transfer in a network. **(B)** Local efficiency *E*_*loc*_, and it measures how efficient communication is among the first neighbors of a given node when it is removed. **(C)** $\lambda =L_{p}^{S\,C}/L_{p}^{S\,C}\,L_{p}^{r\,a\,n\,d}$. **(D)** $\gamma =L_{p}^{S\,C}/L_{p}^{r\,a\,n\,d}$. **(E)** Clustering coefficient*C*_*p*_. **(F)** Characterized shortest path length of SC network *L*_*p*_.

**FIGURE 4:**
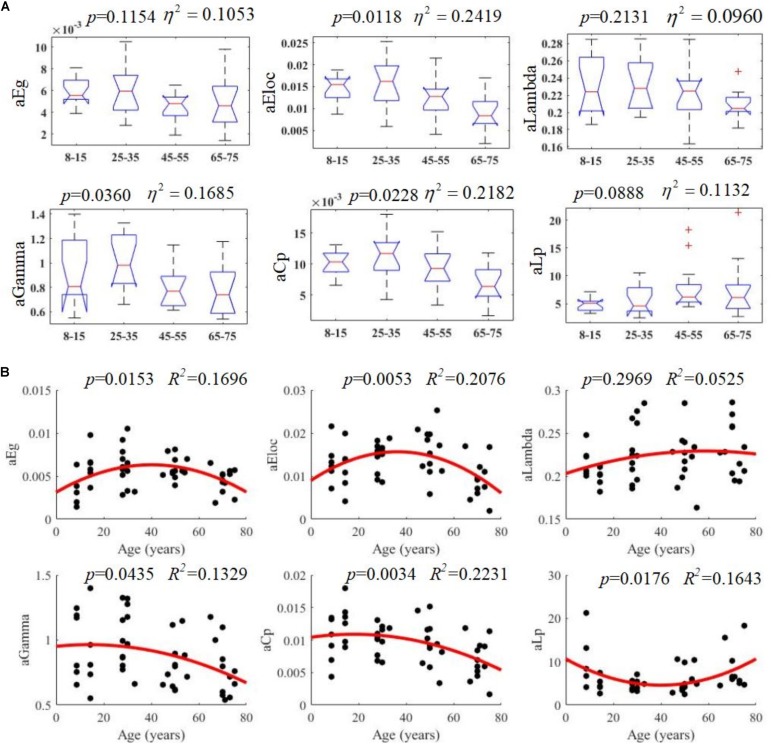
Age-related changes in structural connectivity networks. **(A)** Kruskal–Wallis tests were performed to examine topological differences across age groups, and aEloc, aGamma and aCp can significantly differentiate age groups (*p*-value < 0.05). **(B)** The trajectories of aEg, aEloc, and aCp are statistically significant at *p*-value < 0.05.

All examined groups showed a small-world organization, characterized by γ≫1 and λ > 1 (Figures 3C,D). Compared with other groups, the subjects aged 65–75 years had significantly decreased *E*_*glob*_, *E*_*loc*_, and *C*_*p*_, along with increased *L*_*p*_ (Figures 3A,B,E,F). The descriptive statistics for the AUC values of the thresholded graph metrics are provided in Table 3. Kruskal–Wallis tests indicate that *E*_*loc*_,*C*_*p*_, and γ significantly differentiate the four age groups (*p*-value < 0.05) (Figure 4A). All metrics show a parabolic relationship with age from childhood to old age (Figure 4B), although the *R*^2^ values (5.25–22.31%) were relatively low.

**TABLE 3:** Group-wise comparisons of *AUC values* of global and local network metrics.

Age/y	*aE*_*glob*_	*aE*_*loc*_	*aL*_*p*_	*aC*_*p*_	*a*λ	*a*γ
8-15	0.0059 ± 0.0013	0.0146 ± 0.0034	5.6431 ± 1.9215	0.0101 ± 0.0021	0.2088 ± 0.0168	0.7782 ± 0.2151
25-35	0.0061 ± 0.0024	0.0126 ± 0.0049	4.8403 ± 1.1238	0.0112 ± 0.0037	0.2301 ± 0.0344	1.0163 ± 0.2300
45-55	0.0048 ± 0.0024	0.0158 ± 0.0054	7.5264 ± 1.1707	0.0095 ± 0.0035	0.2314 ± 0.0307	0.8036 ± 0.1806
65-75	0.0045 ± 0.0014	0.0092 ± 0.0045	7.9390 ± 1.4663	0.0070 ± 0.0031	0.2209 ± 0.0305	0.9345 ± 0.2775

*These values were the fitted AUC values (mean ± SD).*

### Hub Regions

**FIGURE 5:**
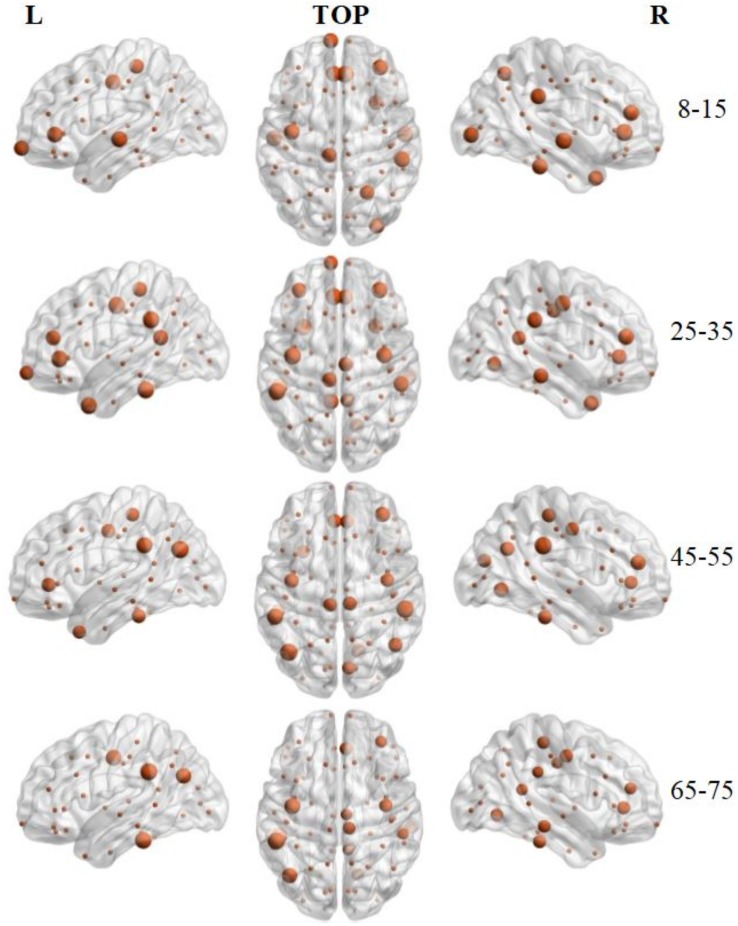
3D distribution of hub regions in the structural connectivity networks of the four age groups. The hub nodes are shown in brown with node sizes indicating their nodal efficiency values. The hub regions were mapped onto the cortical surface at the left, top, and right views. For the index of hub nodes, see Table 4. The brain graphs were visualized by using BrainNet Viewer software (Xia et al., 2013).

Hub regions were identified from the SC networks of each group. The nodes were considered as network hubs if their nodal efficiencies were at least one standard deviation greater than the average nodal efficiency of the network (Cao et al., 2013). The four age groups showed highly similar hub distributions, with core regions mainly in the frontal (region indices: 25, 59, 63) and parietal cortices (region indices: 23, 57, 64, 66) (Desikan et al., 2006), consistent with previous structural connectivity network studies on healthy adults (Sporns, 2013; van den Heuvel and Sporns, 2013). Group-wise analysis using the GRETNA toolbox then revealed that the 65–75 age group had reduced nodal efficiency in the inferior parietal (left hemisphere), inferior temporal (left hemisphere), middle temporal (left hemisphere), precuneus (left and right hemispheres), inferior parietal (right hemisphere), lateral occipital (right hemisphere), and superior parietal (right hemisphere) cortices (Figure 5 and Table 4).

**TABLE 4:** Hub regions distributed in SC networks of the four age groups.

8-15		25-35		45-55		65-75	
Hub regions (index)	*aE*_*nodal*_	Hub regions (index)	a*E*_*nodal*_	Hub regions (index)	*aE*_*nodal*_	Hub regions (index)	*aE*_*nodal*_
16	0.0082	8	0.0103	7	0.0118	7	0.0130
23	0.0087	9	0.0092	8	0.0089	8	0.0143
25	0.0088	16	0.0088	16	0.0087	23	0.0136
29	0.0091	23	0.0105	23	0.0092	30	0.0148
31	0.0084	25	0.0102	25	0.0089	42	0.0105
42	0.0087	26	0.0089	30	0.0107	43	0.0087
44	0.0086	30	0.0104	32	0.0082	46	0.0100
59	0.0097	31	0.0083	38	0.0086	48	0.0100
60	0.0089	32	0.0101	41	0.0092	50	0.0100
62	0.0081	43	0.0082	42	0.0090	56	0.0087
63	0.0093	46	0.0086	46	0.0096	57	0.0107
64	0.0087	48	0.0090	50	0.0089	59	0.0104
66	0.0082	56	0.0085	57	0.0086	60	0.0091
		57	0.0097	59	0.0084	64	0.0083
		59	0.0089	60	0.0099		
		60	0.0097	64	0.0119		
		64	0.0094				
		66	0.0089				

*The nodes were considered brain hubs if their nodal efficiencies were at least 1 SD greater than the average nodal efficiency of the network. The corresponding indexes (see Appendix Table A1) are listed.*

### Backbone Links

**FIGURE 6:**
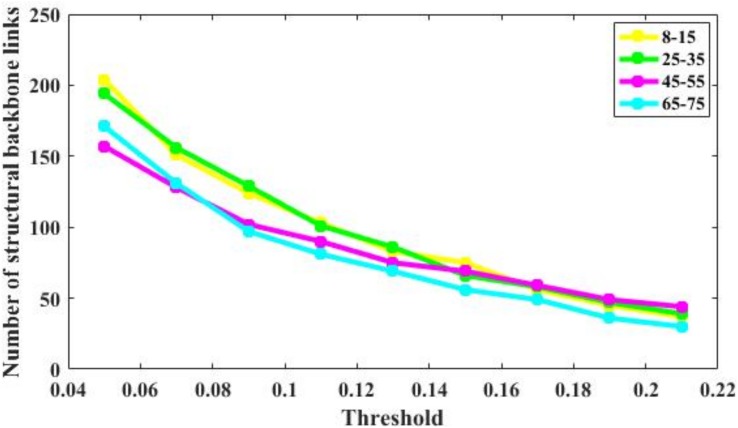
Number of backbone links of the structural connectivity networks of the four age groups. For the 65–75 group, the number of backbone links is decreases significantly. For other groups, there are some minor differences.

**FIGURE 7:**
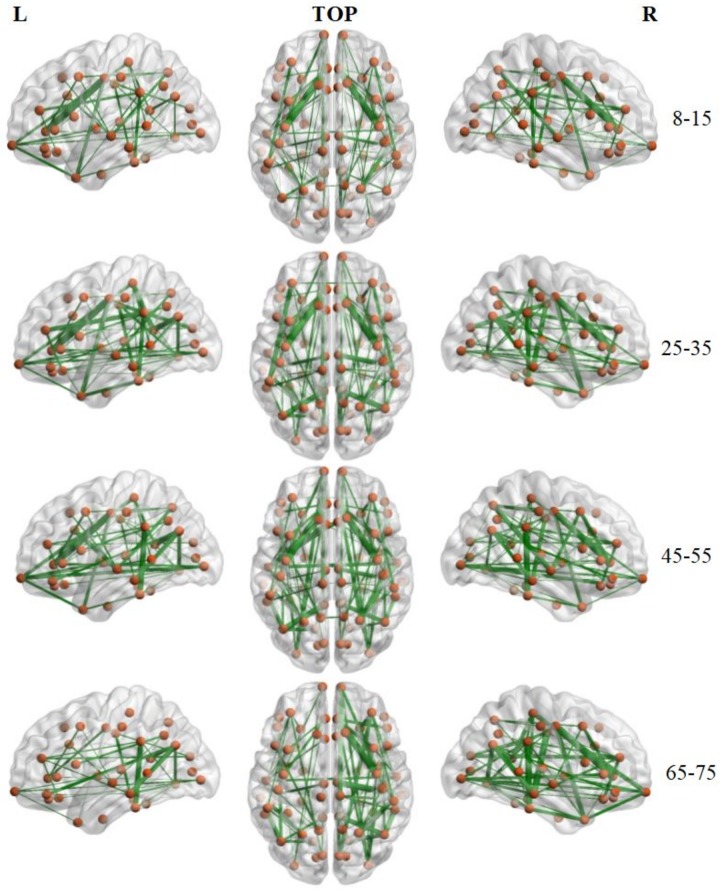
Backbone connections of brain structural connectivity networks of four groups at the strength threshold of 0.21. The links were mapped into the brain at the left, top, and right views. The edge widths represent the connection weights between nodes. The brain graphs were visualized by using BrainNet Viewer software (Xia et al., 2013).

The number of brain structural backbone links in each age group is shown across threshold values in Figure 6, with the specific brain backbone links for each age group at a threshold of 021 shown in Figure 7. Results suggest that the number of backbone links in SC networks of the 65–57 years group significantly decreased as thresholds increased (Figure 6). However, the average backbone connection strengths showed no significant differences between age groups (Table 5).

**TABLE 5:** Connection strength of backbone links at different thresholds.

	>0.05	>0.09	>0.13	>0.17	>0.21
8–15	0.1414 ± 0.0947	0.1900 ± 0.0932	0.2295 ± 0.0904	0.2660 ± 0.0892	0.3066 ± 0.0842
25–5	0.1556 ± 0.1157	0.1993 ± 0.1198	0.2457 ± 0.1225	0.2947 ± 0.1218	0.3467 ± 0.1172
45–55	0.1675 ± 0.1200	0.2207 ± 0.1183	0.2603 ± 0.1143	0.2904 ± 0.1109	0.3254 ± 0.1077
65–75	0.1409 ± 0.1050	0.1956 ± 0.1115	0.2304 ± 0.1150	0.2657 ± 0.1195	0.3154 ± 0.1303

*These values are probability values (mean ± SD) of links in each group.*

## Discussion

The performance of humans on formal cognitive testing and real world cognitive function declines with aging, generally peaking in the 20s and lessening thereafter. Understanding these changes will be critical for gaining insights into the age-associated risk of multiple neurodegenerative disorders and the increased sensitivity of people with aging to various insults, such as infections and brain trauma. Understanding these underlying mechanisms is a first step toward treating or preventing them. Studying structural connectivity may provide vital information for understanding these normal processes of the aging brain and hence its increased vulnerability over the lifespan.

Heretofore, many studies have relied on traditional reconstruction methods that show limited performance when applied to complex fiber populations. In this preliminary study, the performance of a novel Ensemble Average Propagator-based probabilistic tractography method was evaluated as it reconstructed the brain structural connectivity networks of 48 subjects obtained from the HCP and OASIS3 databases. The results suggest a non-linear evolution of age-dependent brain structural connectivity network properties.

The current investigation presents a novel application of the EAP. Previous EAP methods have been used to improve the diagnosis of Parkinson’s disease, stroke detection, and brain tissue assessment (Banerjee et al., 2016; Brusini et al., 2016; Zucchelli et al., 2016); however, this is its first use at addressing structural connectivity. The current probabilistic method was modified from Iturria-Medina et al. (2007), extracting diffusion directions from EAP and avoiding exhaustive searches to identify the strongest paths between different ROIs.

Results obtained through this approach suggest that critical brain structural connectivity properties are conserved throughout the development process, which supports the conclusion that small-world networks are resilient to developmental alteration (Hagmann et al., 2008; Gong et al., 2009; He et al., 2009; Supekar et al., 2010). Despite retaining a small-world topology, however, the 65–75 years subjects showed decreased global efficiency, local efficiency, and increased path length (Figure 3). These findings may reflect structural degeneration due to neuron death or fiber breakdown. These results are supported by a recent study that reported reduced global efficiency in the functional brain networks of older adults (Damoiseaux, 2017). Another study, however, found no differences in global efficiency (Gong et al., 2009). Lack of differences may be ascribed to the use of a diffusion tensor model neural tracking that cannot resolve multiple fiber populations in a DWI voxel.

To examine the topological changes that occur over the lifespan here, a quadratic regression model was fit to the AUC values of each network metric (Figure 4). These results suggest that the lifetime development of structural cortical networks follows a non-linear trajectory. From 7 to 35 years, brain networks strengthen with growth, training, and learning. From 35 to 75 years, however, the network is gradually reduced as overall cortical connectivity declines. These results are mostly consistent with a recent study performed by Zhao et al. (2015), which found a similar non-linear trajectory of structural changes. The present study then investigated the patterns of specific network topological properties. In this way, global efficiency, local efficiency, small-worldness, and cluster coefficients were found to follow negative parabolic trajectories, while characteristic pathway lengths followed a positive parabolic trajectory.

These findings complement the negative parabolic trajectories that were uncovered for the global network properties (Zhao et al., 2015), including network strength, cost, topological efficiency, and robustness. Together, these results suggest that the overall shape of graph network trajectories is consistent and that SC network changes during development and aging are non-linear. These results, then, identify possible structural substrates underlying functional and cognitive changes during development and aging and may be important for separating pathogenic changes from normal aging processes. Some minor differences between these two studies may arise from differences in the dataset, tractography method, and brain parcellation schemes. The current EAP-based probabilistic tractography method has shown to improve fiber tracking accuracy (Descoteaux et al., 2009). Given the scarcity of data, future studies will be required to further corroborate these important findings.

To identify the hub regions, we examined the nodal efficiency of each cortical region. In the 8–15 age group, 13 regions were identified as the hubs by their large nodal efficiency *E*_*nodal*_ values. In the 25–35 and 45–55 age groups, 18 and 16 regions were identified as the hubs, respectively, and 14 regions were identified as hubs in the 65–75 age group (Table 4). Identified hubs were predominately located in regions of the frontal and parietal cortices that connected with multiple other cortical regions (Mesulam, 1998). This dense interconnectivity suggests their pivotal roles in the human structural cortical networks. The findings align with several previous studies, in which these cortical regions were identified as critical nodes in both structural and functional brain networks in humans (Achard et al., 2006; He et al., 2007). Finally, it should be noted that only the 3rd and 4th age groups included data from both the OASIS3 and HCP datasets. Consequently, these groups included subjects who were scanned using different systems with different acquisition parameters. Although the overall data showed the same trends, the effect that these protocol variations may have had on network construction remains to be studied.

Backbone connections were then investigated across age groups. Although all age groups showed decreases in the backbone number as the threshold increased, the number of backbone connections in the 65–75 years group showed a particularly noteworthy reduction (Figure 6). The connections that survived at the highest threshold value were primarily composed of the prefrontal cortex and the insular cortex (Figure 7). These results suggest a strong correlation between the SC wiring within the prefrontal and insular cortex and the behavioral symptoms of old age, such as inattention and amnesia. While the adoption of a lower threshold may provide a more comprehensive snapshot of the structural network, probability-based approaches carry the possibility of identifying spurious nerve fiber connections between regions that are not biologically connected. The use of a high probability threshold may assuage this issue and suggests that present results are not dependent on an arbitrarily chosen threshold. To ensure that spurious connections were removed, a range of thresholds were applied between 0.05 and 0.21 in intervals of 0.02. Links whose connection strength fell below the chosen threshold were excluded from topological analysis ensuring that only strong connections were retained. Despite the tested range, no threshold value caused a significant change in the non-linear patterns of age-dependent brain structural connectivity networks.

The human brain undergoes both macro- and micro-scale structural changes throughout its lifespan (Lebel and Deoni, 2018). The white matter plasticity in response to learning or environmental stimuli can alter the brain’s structural connectivity network, although the magnitude and time course of these changes can vary. Comprehensive lifespan studies provide valuable insight into the processes of macrostructural brain changes and have contributed to our understanding of brain development (Gong et al., 2009; Hagmann et al., 2010; Betzel et al., 2014; Otte et al., 2015; Zhao et al., 2015). The large-scale brain changes during development provide a context for studying white matter changes at the macroscopic level. The current work aimed to characterize normative brain development from 8 to 75 years, emphasizing group-wise comparative studies and statistical techniques to study structural connectivity network development. Previous studies have demonstrated consistent, rapid white matter development over the first 3 years of life, suggesting increased myelination and axonal packing (Huang et al., 2013). Results here clearly demonstrates that the structural connectivity network continually strengthens during later childhood, adolescence, and adulthood, due to white matter changes.

There are several limitations to this study. First, this study had a relatively small sample size (*N* = 48) and results should be interpreted in that context. Nevertheless, the parabolic patterns of functional changes observed using the novel EAP method are promising, suggesting that an EAP-based method is a reliable tool for future studies. These preliminary findings will require further corroboration from future studies with larger-sample sizes. Second, a probabilistic tractography method based on EAP was used to construct whole-brain neural connectivity, which is more capable of tracking complicated fiber tracts than deterministic tractography methods (Behrens et al., 2007). While this provides a better reconstruction of the brain structural network, the reliance on a probabilistic method allows for connections to be drawn between regions that are not biologically linked. Spurious connections are likely to show limited connection strength, however, so the adoption of a high probability threshold should minimize this issue, and the number of remaining connections further suggests that results were not dependent on an arbitrarily chosen threshold.

Third, the whole-brain structural connectivity networks were only derived from DWI data. The brain networks can also be studied using both structural and functional MRI data. The combination of multimodal neuroimaging techniques should add considerably to our understandings of how age-linked structural disruptions in neuronal circuits are associated with functional alterations. Lastly, subjects from the different datasets (HCP and OASIS3) were collected using different scanners and different acquisition protocols, the effects of which are currently unknown and require further study. Although T1-weighted images were denoised through a non-local SVD method (Wu et al., 2019b), alleviating the possible influences of white matter hyperintensities (WMH) on the WM segmentation, future investigations will be necessary to understand the extent to which WMHs could affect EAP calculation (Popescu et al., 2014; Valverde et al., 2014; Prados et al., 2016). Finally, several studies have suggested that the graphic metrics of whole-brain structural connectivity networks are heavily dependent on the resolution of their cortex parcellation (Wang et al., 2009). In the future, different parcellation schemes should be used in conjunction to provide a holistic investigation of topological SC network features.

## Conclusion

Non-linear parabolic patterns of age-dependent brain structural connectivity network properties were observed across the age ranges of 8–75 years using a probabilistic Ensemble Average Propagator-based tractography method. This novel method provided a reliable method to construct macroscale structural connectivity networks that capture age-associated changes in brain structure. This study reveals new insights into age related changes in cognitive function that are observed clinically and starts the process of helping us understand where to focus our efforts at understanding the very important, age-related brain changes that markedly increase vulnerability to neurodegenerative disorders. Understanding age-related vulnerability, in turn, will help us design methods to reduce these age related risks.

## Data Availability Statement

The datasets generated for this study are available on request to the corresponding author.

## Ethics Statement

Ethical review and approval was not required for the study on human participants in accordance with the local legislation and institutional requirements. The patients/participants provided their written informed consent to participate in this study.

## Author Contributions

ZW conducted the data analysis, result summarization, and manuscript drafting. YP contributed to study design, result preparation, and manuscript revising. SS and PS contributed to study design and manuscript revising with clinical knowledge. YZ contributed to study design, result review, manuscript revising, and finalization.

## Conflict of Interest

The authors declare that the research was conducted in the absence of any commercial or financial relationships that could be construed as a potential conflict of interest.

## Figures and Tables

**TABLE A1:** Cerebral cortex was parcellated into 68 functional areas, 34 for each hemisphere.

Index	Region	Index	Region	Index	Region	Index	Region
1	lh.bankssts	18	lh.parahippocampal	35	rh.bankssts	52	rh.parahippocampal
2	lh.caudalanteriorcingulate	19	lh.parsopercularis	36	rh.caudalanteriorcingulate	53	rh.parsopercularis
3	lh.caudalmiddlefrontal	20	lh.parsorbitalis	37	rh.caudalmiddlefrontal	54	rh.parsorbitalis
4	lh.cuneus	21	lh.parstriangularis	38	rh.cuneus	55	rh.parstriangularis
5	lh.entorhinal	22	lh.pericalcarine	39	rh.entorhinal	56	rh.pericalcarine
6	lh.frontalpole	23	lh.postcentral	40	rh.frontalpole	57	rh.postcentral
7	lh.fusiform	24	lh.posteriorcingulate	41	rh.fusiform	58	rh.posteriorcingulate
8	lh.inferiorparietal	25	lh.precentral	42	rh.inferiorparietal	59	rh.precentral
9	lh.inferiortemporal	26	lh.precuneus	43	rh.inferiortemporal	60	rh.precuneus
10	lh.insula	27	lh.rostralanteriorcingulate	44	rh.insula	61	rh.rostralanteriorcingulate
11	lh.isthmuscingulate	28	lh.rostralmiddlefrontal	45	rh.isthmuscingulate	62	rh.rostralmiddlefrontal
12	lh.lateraloccipital	29	lh.superiorfrontal	46	rh.lateraloccipital	63	rh.superiorfrontal
13	lh.lateralorbitofrontal	30	lh.superiorparietal	47	rh.lateralorbitofrontal	64	rh.superiorparietal
14	lh.lingual	31	lh.superiortemporal	48	rh.lingual	65	rh.superiortemporal
15	lh.medialorbitofrontal	32	lh.supramarginal	49	rh.medialorbitofrontal	66	rh.supramarginal
16	lh.middletemporal	33	lh.temporalpole	50	rh.middletemporal	67	rh.temporalpole
17	lh.paracentral	34	lh.transversetemporal	51	rh.paracentral	68	rh.transversetemporal

*The prefix of lh indicates left hemisphere, and rh represents right hemisphere. The numbers are the indexes of function regions.*

## Footnotes

1 www.oasis-brains.org

2 http://www.freesurfer.net/fswiki/DownloadAndInstall

## Appendix

The cerebral cortex was parcellated into 68 functional areas, 34 for each hemisphere. The indexes and their corresponding names of function regions are provided in Appendix Table A1.
